# Acute Transverse Myelitis Following COVID-19 Vaccination

**DOI:** 10.3390/vaccines9091008

**Published:** 2021-09-10

**Authors:** Jhih-Jian Gao, Hung-Pin Tseng, Chun-Liang Lin, Jr-Shiang Shiu, Ming-Hsun Lee, Ching-Hsiung Liu

**Affiliations:** 1 Department of Neurology, Lotung Poh-Ai Hospital, Ilan 26546, Taiwan; c087001@mail.pohai.org.tw (J.-J.G.); 1913@mail.pohai.org.tw (H.-P.T.); c923001@mail.pohai.org.tw (C.-L.L.); 2 Department of Emergency Medicine, Lotung Poh-Ai Hospital, Ilan 26546, Taiwan; mrblack@mail.pohai.org.tw; 3 Department of Radiology, Lotung Poh-Ai Hospital, Ilan 26546, Taiwan; 985003@mail.pohai.org.tw

**Keywords:** myelitis, LETM, COVID-19, COVID-19 vaccination, mRNA vaccine, vitamin B12

## Abstract

An increasing number of people are undergoing vaccination for COVID-19 because of the ongoing pandemic. The newly developed, genetically engineered mRNA vaccines are critical for controlling the epidemic disease. However, major adverse effects, including neuroimmunological disorders, are being attributed to this vaccine. For instance, several cases of acute transverse myelitis (ATM) after COVID-19 vaccination have been reported in clinical trials. Here, we report an exceedingly rare case of longitudinally extensive transverse myelitis (LETM), a rare subtype of ATM involving three or more vertebral segments, that occurred shortly after vaccination with the Moderna COVID-19 (mRNA-1273) vaccine, with a comorbidity of vitamin B12 deficiency. The findings of subsequent investigations suggest the possibility that autoimmune responses are triggered by the reactions between anti-SARS-CoV-2 spike protein antibodies and tissue proteins, as well as the interaction between spike proteins and angiotensin-converting enzyme 2 receptors.

## 1. Introduction

Acute transverse myelitis (ATM) is a disease characterized by weakness, sensory disturbances, and bladder and bowel dysfunction. A rare subtype of ATM, longitudinally extensive transverse myelitis (LETM), refers to the inflammation of the spinal cord, involving three or more vertebral segments [1,2]. As the disease progresses, it may cause severe disability. Patients with ATM are categorized as falling under parainfectious, multiple sclerosis, spinal cord ischemia, or idiopathic causes [3].

Cases of ATM after vaccination were also reported on post-vaccination days 5–28 [4]. Although ATM is a sporadic neurological disorder associated with the COVID-19 pandemic globally, ATM following COVID-19 vaccination is rare. As the adoption of COVID-19 vaccines became prevalent worldwide, reports of their adverse effects began to emerge. The American Neurological Association (ANA) investigated the major neurological complications of COVID-19 vaccination, including tremors, diplopia, tinnitus, dysphonia, seizures, the reactivation of herpes zoster, transverse myelitis, stroke, Bell’s palsy, acute disseminated encephalomyelitis, and Guillain–Barré syndrome [5]. Here, we report an exceedingly rare case of LETM that occurred shortly after vaccination with the Moderna COVID-19 (mRNA-1273) vaccine.

## 2. Case Presentation

**Figure 1:**
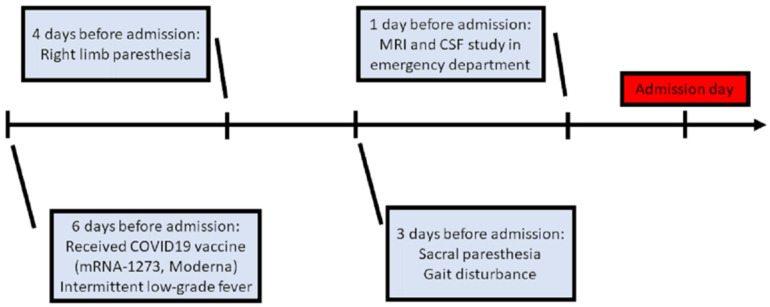
Clinical course before admission. (MRI: magnetic resonance imaging; CSF: cerebrospinal fluid).

A 76-year-old female was admitted to our neurological ward with presentations of unsteadiness and abnormal sensation in the limbs, predominantly on the right side. There were no major significant comorbidities aside from hypertension and right-sided hearing impairment. Six days before admission, she had received COVID-19 vaccine (mRNA-1273, Moderna) in the morning (9 a.m.), after which she experienced intermittent low-grade fever (approximately 37–38 °C) in the evening (4 p.m.). Additionally, she was found to have right upper limb paresthesia that extended from the distal to the proximal limb areas, and to the right lower limb on post-vaccination day 2. She had no symptoms of mental deterioration, headache, neck rigidity, vertigo, nausea, vomiting, fecal or urinary disturbance. There was no history of vegetarianism, gastric surgery, or nitrous oxide exposure. However, progressive gait disturbance and sacral paresthesia occurred on post-vaccination day 3 (Figure 1).

On examination, she exhibited relatively good muscle strength, but also decreased proprioceptive sensation below the right T4 dermatome. The patient also exhibited impairment in joint position sense and thermal analgesia in the right limbs. The deep tendon reflex of the right limbs was relatively brisk. The Babinski sign showed a right extensor plantar response.

**Figure 2:**
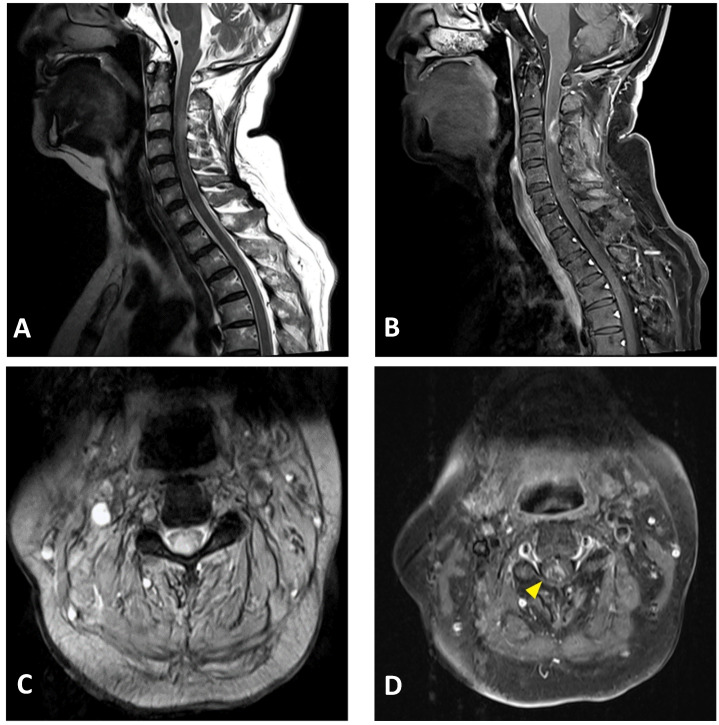
Cervical MRI images of a 76-year-old female with longitudinally extensive transverse myelitis: (**A**) sagittal T2-weighted image showing hyperintensity in the cervical cord at the C2–C5 levels; (**B**) sagittal T1-weighted image with contrast showing ring enhancement in the cervical cord at the C3 level; (**C**) axial T2-weighted image showing extensive hyperintensity in the cervical cord at the C3 level; (**D**) axial T1-weighted image with contrast showing right-sided enhancement in the cervical cord at the C3 level (arrowhead).

A neuroimaging study of the brain and cervical cord was performed on post-vaccination day 5, using magnetic resonance imaging (MRI). C-spine MRI revealed extensive intramedullary hyperintensity in the cervical cord at the C2–C5 levels on T2-weighted images, and at the C3 level with T1 ring enhancement of the cervical cord (Figure 2). Brain MRI and magnetic resonance angiography showed no abnormal findings. Cerebrospinal fluid (CSF) analysis showed mild pleocytosis (15/µL) with neutrophil predominance (73%) and increased protein levels (57.2 mg/dL; normal limit: 15–45 mg/dL). CSF rapid plasma reagin (RPR), treponema pallidum hemagglutination (TPPA), human immunodeficiency virus (HIV), and cytology were all negative. The multiplex polymerase chain reaction (PCR) assay FilmArray meningitis/encephalitis panel did not detect pathogens such as bacteria, viruses, and fungi in the central nervous system. Rheumatoid factor and antinuclear antibody levels were within normal limits. Serum anti-aquaporin 4 (AQP4) antibodies were negative. Somatosensory evoked potential and nerve conduction studies revealed bilateral peroneal neuropathy. The motor and visual evoked potentials were normal. Brainstem auditory evoked potentials showed right sensorineural hearing impairment. The visual evoked potential study revealed a non-significant finding. The CSF was negative for the oligoclonal band. Additionally, analyses for detecting connective tissue disorders and vasculitis profiles revealed no abnormalities (Table 1).

**Figure 3:**
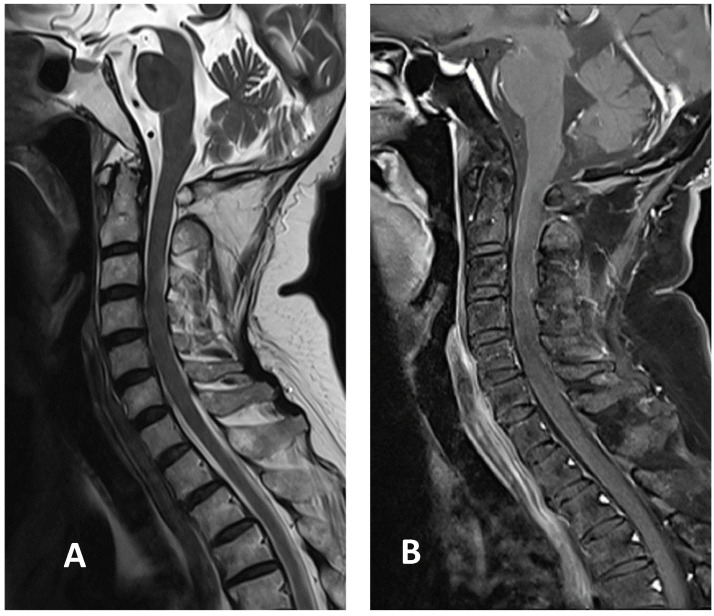
Cervical MRI images two weeks after discharge: (**A**) sagittal T2-weighted image showing the decreased size of hyperintensity in the cervical cord at the C3 level; (**B**) sagittal T1-weighted image with contrast showing minimal enhancement in the cervical cord at the C3 level.

Based on the Brighton case definition for myelitis, the patient was diagnosed with LETM with level 2 diagnostic certainty [6]. Pulse therapy with intravenous methylprednisolone (1 g/day for five days) was initiated after admission. The patient showed improvement in limb and sacral paresthesia symptoms after the pulse therapy, following which, oral prednisolone (60 mg/day) was administered. The examinations also revealed a deficiency in vitamin B12 levels, at 131 pg/mL. Therefore, hydroxocobalamin (1 mg/day) was included in the regimen to compensate for the deficiency. Since the patient gradually regained sensation in the limbs affected by paresthesia, prednisolone was slowly tapered. Her gait also improved following rehabilitation. The patient was discharged two weeks after admission with oral prednisolone, 15 mg twice daily. It was tapered to 10 mg twice daily 1 week later during outpatient department treatment. A follow-up cervical MRI two weeks after discharge showed that the T2 hyperintensity had decreased in size, with minimal enhancement in the T1-weighted image (Figure 3). Prednisolone was tapered to 5 mg three times daily. It was further tapered to 10 mg once daily, four weeks after discharge. Her gait improved without the assistance of a cane.

## 3. Discussion

We present the first case of myelitis (LETM subtype) that acutely manifested as a unilateral sensory deficit following the first dose of an mRNA COVID-19 vaccine. LETM indicates a lesion that extends over three or more vertebral segments, as defined by a spinal MRI [2,7]. It is frequently associated with other autoimmune diseases, such as neuromyelitis optica (NMO) [7]. There were no anti-AQP4 antibodies in our patient, which ruled out the diagnosis of AQP4-Antibody-Positive NMO spectrum disease.

The most common neurological symptoms that occur after the COVID-19 vaccination are transient dizziness, headache, pain, muscle spasms, myalgia, and paresthesia [5]. Transverse myelitis after COVID-19 vaccination is rare (9 cases/51,755,447 dosages), according to ANA investigations [5]. A few cases of ATM were reported following vaccination with the recombinant ChAdOX1 nCoV-19 (AZD1222, Oxford/AstraZeneca, COVISHIELD^TM^) vaccine [8,9,10]. As for mRNA COVID-19 vaccines, only one case has been reported online: a 63-year-old man who presented with distal cord myelitis following the second dose of the Moderna mRNA-1273 vaccine [11].

The temporal relationship between vaccination and ATM in our case was reasonable (48 h post-vaccination). A total of 119 cases of transverse myelitis that developed after vaccination were monitored during the period between 1985 and 2017 (29 men and 90 women; mean age: 32 years; age range: 12–61 years; mode 28 years) [12]. With regard to cases that occurred within the first six weeks after vaccination, the data suggest that the association between vaccination and some of these cases may not be coincidental [12]. The onset of neurological symptoms in our patient, at two days after COVID-19 vaccination, was shorter than that reported in previous case reports [9,10]. However, it was longer than that in the other reported case of myelitis related to the Moderna mRNA-1273 vaccine (onset of symptoms after 17 h) [11].

Fever and pleocytosis after vaccination were observed in the present case. The clinical picture is consistent with the diagnosis of myelitis, with level 2 diagnostic certainty. According to the case definitions and guidelines for collection, analysis, and presentation of immunization safety data [6], for a level 1 diagnostic certainty of myelitis, acute cord inflammation should be demonstrated by histopathology. Level 2 diagnostic certainty requires the presence of symptomatic myelopathy in addition to at least two of the following indicators: fever up to 38 °C, a CSF pleocytosis of >5 WBC/mm^3^, and neuroimaging findings indicating acute inflammation/demyelination of the spinal cord.

Post-vaccination myelitis has been reported in sporadic cases [13,14]. Infectious agents and vaccine adjuvants may evoke autoimmunity in similar ways, for instance, by molecular mimicry, epitope spreading, upregulation of cytokines, and polyclonal activation of B and T lymphocytes, which may induce immune reactions associated with ATM [13]. It is believed that the pathogenesis caused by adenoviral vaccines might be related to the use of chimpanzee adenovirus vectors [8,15]. However, the Moderna COVID-19 (mRNA-1273) vaccine is composed of an mRNA vaccine encoding the pre-fusion spike protein encapsulated in lipid nanoparticles, with no adjuvants [16]. Therefore, other mechanisms might be involved in the development of autoimmunity in such cases. For instance, a study suggested that the immunological reaction between the SARS-CoV-2 spike protein antibody and tissue proteins, such as myelin basic protein, may be a plausible cause for the occurrence of demyelinating autoimmune diseases [17]. Furthermore, the inflammatory response triggered by the interaction between spike proteins and angiotensin-converting enzyme 2 (ACE2) receptors present in the endothelial cells of the blood–brain barrier or spinal neurons may be another possible mechanism of demyelination [18,19].

Post-vaccination ATM-superimposed vitamin B12 deficiency was also observed in our patient. Nevertheless, the evidence suggested that the cervical lesions are more likely due to acute myelitis than subacute combined degeneration (SCD) [20]. In addition, the onset of sensory and gait disturbances was acute and not subacute. Furthermore, cervical MRI showed an eccentric (right-sided), ring-enhancing lesion, favoring a diagnosis of transverse myelitis, and not the typical inverted V sign of SCD [21,22]. Contrast enhancement of the spinal cord in our patient was also not typical of SCD [23]. Finally, the CSF showed pleocytosis, which suggested an inflammatory response and not SCD [20]. Since vitamin B12 is known as a cofactor of myelin formation as well as immunomodulation and neurotropism [24,25,26] while it could be a coincidental finding, the confounding role of vitamin B12 should not be underestimated.

Anti-myelin oligodendrocyte glycoprotein (MOG) antibodies were not examined in the patient due to our limitations. Because the MOG antibodies test is not including in Taiwan’s national health insurance system, it cannot be afforded in testing for economic reasons. There were few cases with post-vaccination encephalomyelitis that were related to MOG antibodies [27,28]. As for our patient, there was no evidence of encephalitis. However, MOG antibody related disorders cannot be ruled out in our patient.

## 4. Conclusions

Here, we present a case of LETM, a scarcely reported disorder, that occurred shortly after receiving the first dose of an mRNA-based COVID-19 vaccine, with comorbid vitamin B12 deficiency. However, since LETM has rarely been associated with vaccination, the association of LETM with the Moderna COVID-19 (mRNA-1273) vaccine is still uncertain. There is a possibility that autoimmune reactions were triggered by the reaction between the SARS-CoV-2 spike protein antibody with tissue proteins, and the interaction between spike proteins and ACE2 receptors, as has been discussed earlier. However, the confounding role of vitamin B12 deficiency is unclear, and should be investigated in further reported cases and immunological studies.

## Figures and Tables

**Table 1:** Laboratory profile.

Parameter [Unit]	Result	Reference Value
WBC [10^9^/L]	4.60	4.50–11.00
Hgb [g/dL]	12.0	12.00–16.0
Platelet [10^9^/L]	296	150–400
MCV [fL]	113.7	80.0–96.0
WBC CSF [/µL]	15	
RBC CSF [µL]	73	
Lymphocyte CSF [%]	27	28–96
Neutrophil CSF [%]	73	
Lactate CSF [mmol/L]	2.7	<2.8
Protein CSF [mg/dL]	57.2	15.0–45.0
Glucose CSF [mg/dL]	71	40–70
Glucose serum [mg/dL]	117	
ESR [mm]	27	0–20
CRP [mg/dL]	0.06	<0.50
Anti-HIV EIA [COI]	0.11	<0.9
TPPA CSF	Negative	Negative
RPR/VDRL CSF	Non-reactive	Non-reactive
Electrophoresis CSF	No oligoclonal band	
Vitamin B12 [pg/mL]	131	Deficient < 211
RF [IU/mL]	<10.0	<14.0
ANA	1:40(−)	
TSH [µIU/mL]	0.18	0.270–4.20
Free T4 [ng/dL]	1.57	0.93–1.70
A-beta2GPI IgG [U/mL]	<1.4	<20.0 U/mL
Anti-cardiolipin IgG [GPL-U/mL]	<1.6	<20.0
ENA anti-SSA [AI]	<0.2	<1.0
ENA anti-SSA 52 [AI]	<0.2	<1.0
ENA anti-SSA 60 [AI]	<0.2	<1.0
ENA anti-SSB [AI]	<0.2	<1.0
cANCA	10× (Negative)	
pANCA	10× (Negative)	
Atypical pANCA	10× (Negative)	
Anti-dsDNA [IU/mL]	<1.0	Negative < 4
TSH receptor Ab [IU/L]	<0.10	Negative < 0.1
Aquaporin 4 antibody	Negative	Negative

WBC: white blood cells count; Hgb: hemoglobin; MCV: mean corpuscular volume; RBC: red blood cells; CSF: cerebrospinal fluid; ESR: erythrocyte sedimentation rate; CRP: C-reactive protein; HIV: human immunodeficiency virus; EIA: enzyme-linked immunoassay; TPPA: treponema pallidum particle agglutination; RPR: rapid plasma regain; VDRL: venereal disease research laboratory; RF: rheumatoid factor; ANA: antinuclear antibody; TSH: thyroid stimulating hormone; T4: thyroxine; GPI: glycoprotein I; IgG: immunoglobin G; ENA: extractable nuclear antigen; SSA: Sjögren’s syndrome-related antigen A; SSB: Sjögren’s syndrome-related antigen B; cANCA: cytoplasmic anti-neutrophil cytoplasmic autoantibodies; pANCA: perinuclear anti-neutrophil cytoplasmic autoantibodies; dsDNA: double stranded deoxyribonucleic acid.

## Data Availability

The data presented in this study are available upon request from the corresponding author.
